# Historical Perspective and Risk of Multiple Neglected Tropical Diseases in Coastal Tanzania: Compositional and Contextual Determinants of Disease Risk

**DOI:** 10.1371/journal.pntd.0003939

**Published:** 2015-08-04

**Authors:** Frederick Ato Armah, Reginald Quansah, Isaac Luginaah, Ratana Chuenpagdee, Herbert Hambati, Gwyn Campbell

**Affiliations:** 1 Environmental Health and Hazards Laboratory, Department of Geography, University of Western Ontario, London, Ontario, Canada; 2 Biological, Environmental & Occupational Health Sciences, School of Public Health College of Health Science, University of Ghana, Legon, Accra, Ghana; 3 Noguchi Memorial Institute for Medical Research, College of Health Science, University of Ghana, Legon, Accra, Ghana; 4 Department of Geography, University of Western Ontario, London, Ontario, Canada; 5 Department of Geography, Memorial University of Newfoundland, St. John's, Newfoundland and Labrador, Canada; 6 Department of Geography, University of Dar es Salaam, Dar es Salaam, Tanzania; 7 Indian Ocean World Centre (IOWC), Montréal, Quebec, Canada; Swiss Tropical and Public Health Institute, SWITZERLAND

## Abstract

**Background:**

In the past decade, research on neglected tropical diseases (NTDs) has intensified in response to the need to enhance community participation in health delivery, establish monitoring and surveillance systems, and integrate existing disease-specific treatment programs to control overlapping NTD burdens and detrimental effects. In this paper, we evaluated the geographical distribution of NTDs in coastal Tanzania.

**Methods and Findings:**

We also assessed the collective (compositional and contextual) factors that currently determine risks to multiple NTDs using a cross sectional survey of 1253 individuals in coastal Tanzania. The results show that the effect size in decreasing order of magnitude for non-binary predictors of NTD risks is as follows: NTD comorbidities > poverty > educational attainment > self-reported household quality of life > ethnicity. The multivariate analysis explained 95% of the variance in the relationship between NTD risks and the theoretically-relevant covariates. Compositional (biosocial and sociocultural) factors explained more variance at the neighbourhood level than at the regional level, whereas contextual factors, such as access to health services and household quality, in districts explained a large proportion of variance at the regional level but individually had modest statistical significance, demonstrating the complex interactions between compositional and contextual factors in generating NTD risks.

**Conclusions:**

NTD risks were inequitably distributed over geographic space, which has several important policy implications. First, it suggests that localities of high burden of NTDs are likely to diminish within statistical averages at higher (regional or national) levels. Second, it indicates that curative or preventive interventions will become more efficient provided they can be focused on the localities, particularly as populations in these localities are likely to be burdened by several NTDs simultaneously, further increasing the imperative of multi-disease interventions.

## Author Summary

Neglected Tropical Diseases (NTDs) are characterized by their high incidence in low-income countries, thus maintaining the disastrous poverty-disease-poverty cycle. Apart from poverty, however, little is known of the magnitude of importance of both compositional and contextual factors in creating disease risk at the local level, although this knowledge is critical to disease control and policy action. In this study, we show that the order of importance of both sets of factors is as follows: NTD comorbidities > poverty > educational attainment > self-reported household quality of life > ethnicity.

## Introduction

Neglected tropical diseases (NTDs)—a group of seventeen core debilitating infectious diseases [1] that mutually reinforce (act as both a cause and effect of) poverty—have increasingly been receiving cumulative policy, public health attention globally. Neglected tropical diseases affect more than 1 billion people [2, 3] predominantly poor populations living in tropical and subtropical climates. NTDs are endemic in 149 countries with differing populations, economies, resources, political and legal arrangements, health regulations, traditions, cultures, climates, infrastructure and geographies [4]. Yet, NTDs frequently cluster together geographically and individuals are often simultaneously afflicted with more than one parasite or infection (comorbidities). We are still in the early stages of appreciating the full extent of the comorbidity that occurs when the neglected tropical diseases are superimposed on the "Big Three" (HIV/AIDS, tuberculosis, and malaria). Integrated approaches are useful in addressing both NTDs and their comorbidities. For instance, lymphatic filariasis and malaria (NTD comorbidity) are both transmitted by mosquitoes thus distribution of bed nets leads to a decline in both diseases. The pathogens of NTDs have exceedingly complex life-cycles, population dynamics, infection processes and epidemiologies, causing diverse diseases and pathologies [1]. Although they are biomedically heterogeneous, the commonality of NTDs is evidenced in their persistence and prevalence in people and communities living in poverty and social exclusion. It is estimated that more than 70% of countries and territories that report the presence of neglected tropical diseases are low-income or lower middle-income economies especially in sub-Saharan Africa [4]. In particular, those living in remote areas are most vulnerable to infections, and their biological and sociocultural consequences [5]. Notwithstanding this, even within low income countries, there are age- and sex-specific differentials in the health outcomes induced by NTDs. For instance, many NTDs disproportionately affect women and children in sub-Saharan Africa [2, 6].

According to Hotez and Kamath [7], the United Republic of Tanzania has the third highest prevalence of two NTDs namely Lymphatic filariasis and Trachoma in sub-Saharan Africa. Hitherto, research on NTDs in many sub-Saharan African countries including Tanzania, which is endemic to at least 7 NTDs (lymphatic filariasis, schistosomiasis, soil-transmitted helminthiasis, onchocerciasis, trachoma, rabies, trypanosomiasis); have disproportionately focused on clinical aspects [8] especially the use of mass drug administration. Madon et al. [9] show that, as at 2011, functioning NTD coordination units had been set up at national, regional and district levels throughout the United Republic of Tanzania providing a 53% geographical coverage for preventive chemotherapy (PCT). Despite the national- and sub-national level priorities on understanding and eliminating NTDs and its associated risks, we know far less about multiple and differential exposures of individuals to NTDs based on population composition and contextual attributes. According to Singer and Bulled [10], although the role of tropical disease syndemics in contributing to the health burden of the poor is significant, they have been largely unrecognized and generally neglected in the existing literature. Syndemics of NTDs, a relatively new theoretical approach, involves a critical examination of the adverse morbidity-enhancing interactions among NTDs and between NTDs and other diseases. A basis for syndemic analysis is the attention to underlying social conditions and relationships, their causes and contexts, as well as the natural and anthropogenic environmental factors that facilitate the clustering and interaction of constituent diseases. This paper adopts this approach in assessing the risk of multiple neglected tropical diseases in coastal Tanzania.

The complex relationships between environmental factors and NTD-induced human health outcomes, taking into account multiple pathways and interactions, should be seen in a broader spatial, socio-economic and cultural context. Since NTD distribution varies systematically by individual characteristics and place-based attributes it is imperative that the scientific research community push for a greater understanding of the differentials in health outcomes associated with NTD aetiology and distribution. This gap in the literature is a fundamental motivation for this paper. The aim of this paper is threefold. First, we attempted to provide a brief historical overview of NTD prevalence over time in Tanzania. Secondly, we assessed the risk of exposure to multiple NTDs in coastal Tanzania. Thirdly, we evaluated the magnitudes of compositional and contextual determinants of NTD risks in coastal Tanzania. Given that not all the NTDs reported in Tanzania can be found in the coastal zone, for the 3rd aim we restricted the statistical analysis to NTDs that only prevail in three coastal regions of Tanzania (our study area). The study area is part of the Indian Ocean world (IOW), an arena of primary geo-political importance in eastern Africa. The specific study regions were selected based on the assumption that NTDs are co-endemic clusters in endemic geographical areas. Therefore, we expected to see more clustering in these areas. Besides, although these geographical areas are contiguous they have large wealth inequalities. To our knowledge, this paper is the first to characterise and quantify differential risks of NTDs based on population composition and contextual attributes. Based on the extant literature, the following hypotheses were formulated to guide the study. First and foremost, NTDs are rooted in poverty [2,5]; poorer individuals will be associated with higher risks of exposures to multiple NTDs and by extension, experience higher NTD risks than their relatively affluent counterparts. Second, neighbourhood disadvantage (expressed in the lack of or limited access to health, social, water and sanitation services) is more strongly associated with NTD risks such that individuals living in poorer neighbourhoods will likely experience higher NTD risks than their counterparts living in more affluent neighbourhoods. Lastly, social inequities and disadvantage make women in developing countries more vulnerable to negative health outcomes [2, 5]. Therefore, women are more exposed to multiple NTDs and higher NTD comorbidities and so experience higher NTD risks than their male counterparts.

### Theoretical Context

The role of space in shaping health inequities (e.g., differential distribution and by extension differential exposure to and risk of NTDs) is of continuing interest in environmental health geography. In studying the role of space in shaping health outcomes, individual-level (compositional) and place-level (contextual) factors have traditionally been identified [11–15]. Usually, the health outcomes experienced by individuals living proximal to one another are more similar to one another than to those of individuals living in distant neighbourhoods. In theory, three plausible explanations account for this observation. First of all, it may purely be that individuals in the same neighbourhood tend to be more similar to one another than to those in other neighbourhoods in terms of predisposing factors such as age, gender and ethnicity, that is, the *composition* effect [13]. Another viable explanation may be that individuals living in the same neighbourhood are exposed to similar local factors that have impacts on their health outcomes (NTD risks), for example proximity to a river where blackfly proliferate or service provision, that is, the *context* effect [13]. It can also be argued that individuals who live in proximity are more likely to engage in the same types of behavior that may have influences on health outcomes–for example behavior of bathing in rivers among adolescents which are affected by peer pressure-the *collective* effect. Since we live in a complex world, in reality all three elements may be present to varying extents in relation to the distribution of neglected tropical diseases. It is, therefore, imperative to include relevant environmental, behavioural and predisposing factors and to recognise the inherent complexity of composition/context/ collective effects [16].

**Fig 1:**
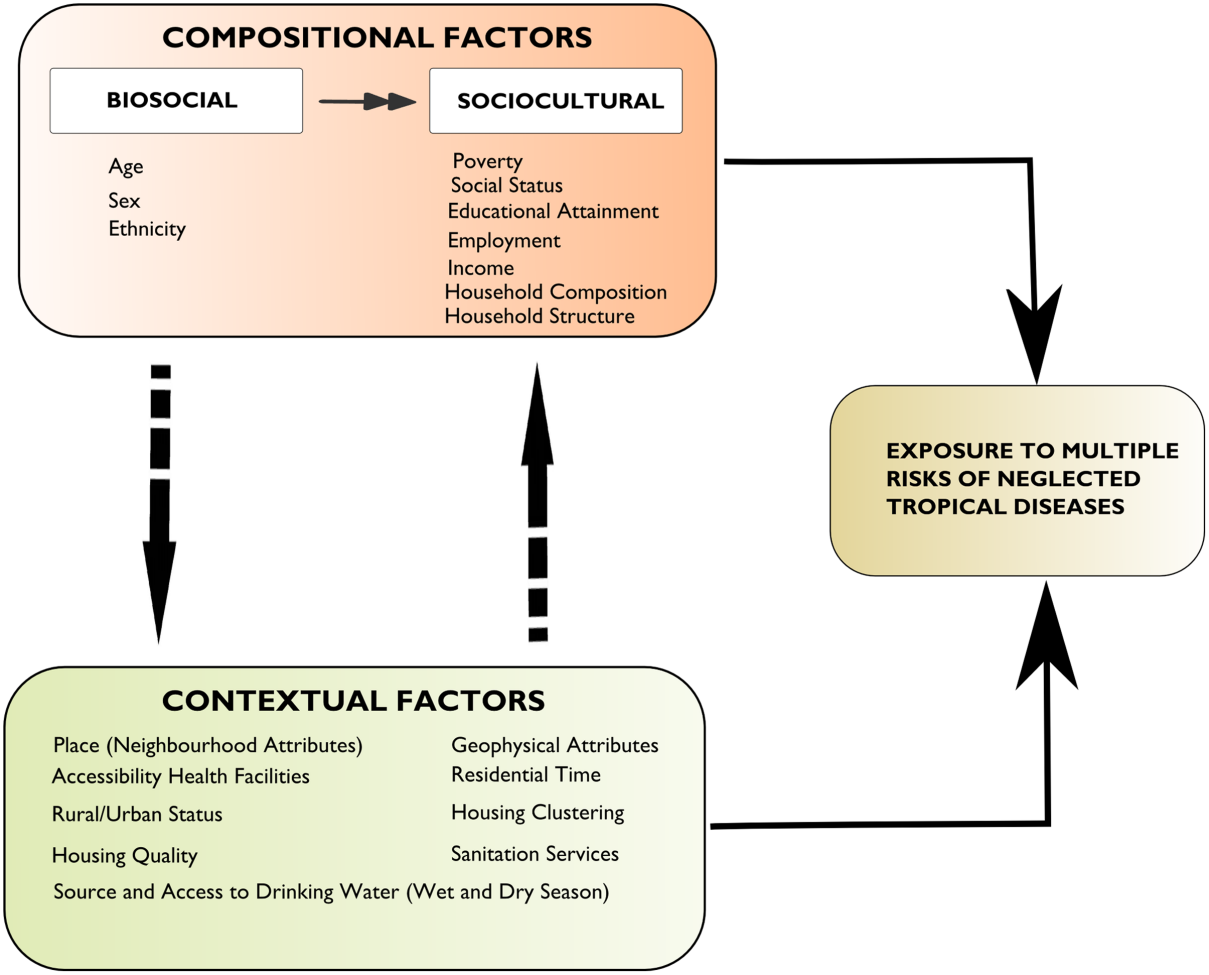
Complex interactions between compositional and contextual factors and exposures to multiple NTD risks.

It is recognized that ‘context’ and ‘place’ vary in time and space. Theoretical and empirical approaches (both qualitative and quantitative) require relational notions of space and place that accepts mutually reinforcing and reciprocal relationships between people and place [11]. Further, scale must be included in the analysis of ‘contexts’ relevant for health. Under this circumstance, place is regarded as complex, socially constructed, unbounded, fluid, and dynamic [11]. It also is multi-scalar, enmeshed in networks, shows social power relations and has cultural meaning [11, 17]. Recent studies also demonstrate that context (e.g. neighbourhoods) is important in determining health outcomes but that compositional factors such as gender, ethnicity, employment status and socio-economic status remain better predictors of inequalities in health [13, 15, 18]. In modelling, the compositional and contextual accounts of health outcomes are usually considered as ‘mutually exclusive, competing, and culturally and historically universal’ [15]. In fact, from a sustainability perspective, this dichotomous framing is rather problematic. Smyth [15] argues that this supposed difference between people and places, composition and context, is rather artificial. In this study, therefore, we move beyond this polar conceptualization and focus on the collective effect of compositional and contextual attributes. According to Cummins et al. [11], a change from empirical research intended to differentiate between contextual and compositional effects to research that focuses on the processes and interactions occurring between places and people and over time is important for understanding health outcomes (e.g. the distribution of NTDs), and is justified. It is in this milieu that this paper should be understood. In Fig 1 we show how compositional and contextual attributes jointly shape exposures to multiple NTD risks.

In Fig 1, we observe that exposures of individuals to multiple health risks associated with NTDs involve complex and dynamic interplay among biological, environmental, and sociopolitical components spanning multiple time intervals and spatial (geographic) scales. In this context, the question is more about what types, in what places, how they contribute and how they can be addressed.

In this framework, we consider NTD risks and health outcomes as emergent properties of complex interactions between humans and their environment. There are several feedback relationships in Fig 1 indicating the multi-factorial nature of both the determinants and the manifestations of NTD health outcomes in coastal populations. For instance, there is a bidirectional linkage between compositional and contextual factors. Similar relationships exist between NTD risks and compositional factors on the one hand and NTD risks and contextual factors, on the other hand. Risks of individuals to NTDs emanate from two mutually-reinforcing factors namely exposure and vulnerability. Predisposing factors to vulnerability include both biosocial and sociocultural dynamics. The latter, together with contextual attributes, also contribute to exposures of individuals to multiple NTD risks. Altogether, this interactivity between variables and processes, which are shaped by multiple factors, warrants a systems approach to addressing exposures and risks to multiple NTDs. A systems approach comprehensively considers all known and measurable aspects of a problem, including feedbacks that cross the boundaries of sub-systems and cut across scales; it acknowledges the nonlinearities and the dynamic nature of underlying processes, uncertainty and surprises [19]. Its potential can be harnessed by policymakers/researchers as they focus more on the social determinants of health when designing NTD interventions (and target interventions).

## Materials and Methods

### Study Area

**Fig 2:**
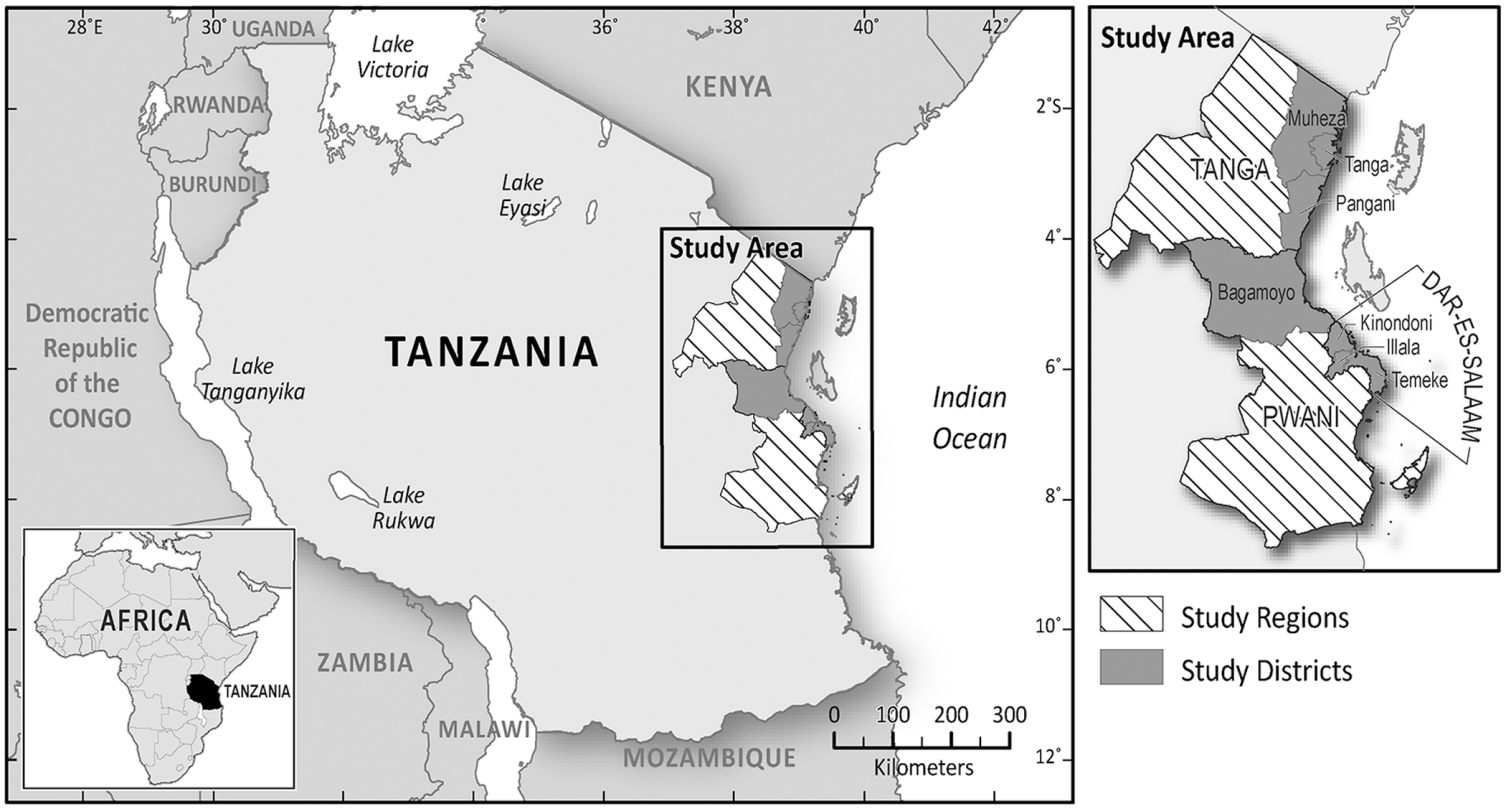
Map of Tanzania showing the study areas.

Tanzania is a coastal country lying between longitude 29° and 49° East and latitude 1° and 12° south of the Equator [20] (Fig 2). The marine waters comprise 64 000 km^2^ as territorial waters and 223 000 km^2^ as offshore waters (EEZ) [21]. Tanzania’s coastline stretches for 800km. It has five coastal regions-Tanga, Pwani, Dar-es-Salaam, Lindi and Mtwara. The five coastal regions cover about 15 percent of the country’s total land area and are home to approximately 25 percent of the country’s population [22]. According to the 2012 Population and Housing census, the total population was 44,928,923 compared to 12,313,469 in 1967 [23], reflecting an annual growth rate of 2.9 percent. The under 15 age group represented 44.1 percent of the population, with 35.5 percent being in the 15–35 age group, 52.2 percent being in the 15–64 age group, and 3.8 percent being older than 64 [23]. Overall Tanzania on average is sparsely populated with population density of 51 persons per square kilometer, lower significant variation exists across regions. The population density varies from 1 person per square kilometre in arid regions to 51 per square kilometre in the mainland's well-watered highlands to 134 per square kilometre in Zanzibar [24]. The population density for the Dar es Salaam region is 3,133 persons per km^2^ (the most densely populated) and that of Lindi is only 13.1 persons per km^2^ [23]. This suggests wide disparities in population density across regions. This study specifically focuses on Dar-es-Salaam, Pwani and Tanga. The 3 coastal regions selected for analysis were chosen for two main reasons. First, the three regions are of historical significance to the Indian Ocean World project. Second, these regions were selected because of the 5 regions, they are the most ethnically diverse (that is, representative of the different geographical locations) and thus, had better prospects of providing heterogeneous survey responses. Dar es Salaam is the capital of the Dar es Salaam Region, which is one of Tanzania's 26 administrative regions. The Dar es Salaam Region consists of three local government areas or administrative districts: Kinondoni to the north, Ilala in the center of the region, and Temeke to the south. Pwani (coast) is the 21^st^ most densely populated region. It is bordered to the north by the Tanga Region, to the east by the Dar es Salaam Region and the Indian Ocean, to the south by the Lindi Region, and to the west by the Morogoro Region. Tanga region has a population of 2,045,205 [24]. It is bordered by Kenya and Kilimanjaro Region to the north; Manyara Region to the west; and Morogoro and Pwani regions to the south. Its eastern border is formed by the Indian Ocean.

Seven districts namely Kinondoni, Temeke and Illala (in Dar-es-Salaam region), Bagamoyo (in Pwani region), and Tanga Town, Muheza and Pangani (in Tanga region) were considered in this study. According to the Tanzania National Bureau of Statistics [23], Kinondoni municipality has a population of 1,775,049 and density of 3302.8 inhabitants per km^2^. Illala municipality has a population of 1,220,611 and density of 3344.4 inhabitants/km^2^ and Temeke municipality has population of 1,368,881 and density of 1878.5 inhabitants/km^2^. The population and density of Bagamoyo are 311,740 and 36.8 inhabitants/km^2^ respectively whereas that of Muheza is 204,461 and 136.5 inhabitants/km^2^, respectively [23]. Tanga town has a population of 237,332 and density of 458.2 inhabitants per km^2^ and Pangani district has a population of 54,025 and density of 30.8 inhabitants per km^2^. The numbers of participations from each of seven districts were as follows Kinondoni (360), Temeke (101), Illala (140), Bagamoyo (301), Tanga Town (129), Muheza (101) and Pangani (121).

### Ethics Statement

The study was approved by the Research Ethics Board of the University of Western Ontario, Canada. Research approval was also granted by the Commission on Science and Technology (COSTECH) in Tanzania. Written informed consent was obtained from participant prior to commencement of the study.

### Data Collection

In addressing the first aim (a brief historical perspective on NTD prevalence and distribution across Tanzania) we conducted a literature search on the NTDs that have been reported in Tanzania since the 19^th^ century. Using the country- and disease-specific query, we searched the Global Infectious Diseases and Epidemiology Network (GIDEON) database (http://www.gideononline.com/) and the global NTD (GNTD) database (http://www.gntd.org/). We further obtained secondary data from the Tanzania National Institute for Medical Research (NIMR). For triangulation, we interviewed experts from the national office of the Tanzania NTD Control Program (TZNTDCP) as well as the Ministry of Health and Social Welfare’s (MOHSW) integrated NTD program.

In addressing the second objective (assessment of the risk of exposure to multiple NTDs along the coastline of Tanzania) we examined most-at-risk populations in coastal Tanzania and NTD profiles at the community level during the last five and ten years. This information was complemented with data from a cross sectional survey.

The third aim was achieved by conducting a cross sectional survey with 1253 individuals in three regions (Dar es Salaam, Tanga, and Pwani) along the coastline of Tanzania. The survey data were collected between March and September 2013. The study population included male (606) and female (647) participants between the ages of 18 and 70+ years. The study used multistage sampling to obtain representative estimates of the population of residents of the three regions. Within each region, a list of villages based on the 2012 Population and Housing Census was divided further into households. The list of villages was also divided into clusters ensuring that each cluster would provide adequate numbers of eligible respondents to be included in the survey. This approach both corrects for sampling bias and weights the cases to match census percentages of males and females of various age groups and by ethnicity. The enumeration areas (EAs) and their total number of households were listed geographically by urban and rural areas. Where EAs did not include the minimum number of households, geographically adjacent EAs were amalgamated to yield sufficient households. This provided the frame for selecting the clusters to be included in the survey according to a stratified systematic sampling technique in which the probability for the selection of any cluster was proportional to its size. A sampling interval was calculated by dividing the total number households by the number of clusters. A random number between 1 and the sampling interval was computer generated. The EA in which the random number fell was identified as the first selected cluster. The sampling interval was applied to that number and then progressively until the 20 (urban) and 15 (rural) clusters were identified. These clusters made up the sample for the survey. Households were randomly selected from these clusters for interview.

### Measures

#### Outcome variable

Based on whether the specific NTD had been reported for at least two consecutive years in the past 5 years or not in the communities of the most-at-risk populations, six focal NTDs were considered in this paper: cholera, schistosomiasis (bilharzia), onchocerciasis (river blindness), trachoma (granular conjunctivitis), hookworm and whipworm. For the six NTDs, each of the 1253 individuals in the survey were either assigned a value of 1 (exposed) or 0 (unexposed) depending on whether the specific NTD had been reported in the neighbourhood at least once in the past 5 years or not. Neighbourhood is used to describe an area surrounding a local institution patronized by residents, such as a health centre. It can also be defined by a political ward or precinct. The concept of neighborhood includes both geographic (place-oriented) and social (people-oriented) components. City planning departments often designate neighbourhood boundaries along census tract boundaries. And, in fact, community residents quite frequently have a very different mental map of their neighbourhood than the officially designated neighborhood areas used by planners and policymakers. In the context of this paper, neighbourhoods refer specifically to administrative wards. From the foregoing, we emphasize that the statistical relationship is between survey data on social determinants and reported cases of certain NTDs at the neighbourhood level. The same point applies to the use of the term co-morbidities, which we are assessing at a geographic (and not individual) level. It is in this context that the term *exposure* should be understood.

The exposure to multiple NTDs was calculated as the algebraic sum of the exposure to each of the six NTDs. Several assumptions underlie this exposure summation technique. It assumes homogeneous exposure within the same neighbourhood; thus, likely intra-neighbourhood differentials in exposure to NTDs were considered as random. It also assumes independence of the exposure to each of the six NTDs. It also assumes that the magnitude of an adverse NTD-related health effect will be proportional to the sum of the ratios of the exposures. If these assumptions are incorrect, over- or under-estimation of the actual multiple-NTD risk could result.

#### NTD Risk = Function (Exposure, Vulnerabilities)

Vulnerabilities of individuals to NTDs were assigned based on their responses to 10 objective health measures in the survey. These included whether or not respondents had been diagnosed with malaria in the past 12 months, diagnosed with HIV, diagnosed with pneumonia, hepatitis, skin conditions or tuberculosis in the past 5 years. Also, respondents were asked whether they had been diagnosed with heart disease, cancer, hypertension or diabetes. In each case, respondents who answered in the affirmative were each assigned a value of 1 otherwise 0 was assigned. We hypothesized that higher number of affirmatives corresponds to more vulnerability of the respondent. Apart from their use as vulnerability indicators, the 10 objective health measures also constituted an additive score for NTD comorbidity.

For each NTD:

Risk = ∑Respondents Exposure scores  × Respondents Vulnerability scores 

Assuming uniform weight of each NTD based on severity and importance, then

$$\text{Total NTD Risk = Function }\left({\text{Risk}}_{\text{cholera}}{\text{, Risk}}_{\text{schistosomiasis}}{\text{, Risk}}_{\text{onchocerciasis}}{\text{, Risk}}_{\text{trachoma}}{\text{, Risk}}_{\text{hookworm}}{\text{, Risk}}_{\text{whipworm}}\right)\text{.}$$

The set of NTD risks was then constituted into a composite score. Cronbach’s alpha test of reliability computed for the six NTD risk items was 0.83 showing good internal consistency. Total Exposure NTD Risk was calculated using principal component and factor analyses. Only one factor had an Eigen value >1 and was retained (all the components loaded on a single construct). This factor explained approximately 90% of the variance in the 6 NTD risk scores.

#### Compositional and contextual factors

Theoretically relevant compositional (biosocial and sociocultural) factors used in this study include gender, age of respondents, ethnicity, poverty, educational attainment, marital status, and employment status. The contextual factors include household quality, exposure to single or multiple NTDs, NTD comorbidities, access to health services, rural/urban status, access to good drinking water during the dry season, and access to good drinking water during the wet season.

### Statistical Analysis

Inferential and multivariate techniques were applied to examine associations between NTD risks and theoretically relevant compositional and contextual factors variables using STATA 13SE software. The Ordinary Least Squares technique was employed for the analysis. Analyses were preceded by diagnostic tests to establish whether variables met the assumptions of the regression model. Bivariate analysis was initially performed to examine zero-order correlations between the NTD risks and theoretically-relevant independent variables all of which were significant and in the expected direction, thus supporting good construct validity. Further, multivariate models were estimated to explore the net effects of the predictor variables using the stepwise selection approach. The relative quality of candidate multivariate models (both OLS and ordinal logit based on categorized NTD risks) was tested using Akaike Information Criterion (AIC). After comparison, the best model was chosen based on parsimony, our working hypotheses, and strength of evidence. For analytical purposes, the unstandardized regression coefficients were estimated although standardized regression coefficients were used as indices of effect sizes of the non-binary predictors. Positive coefficients for any of the predictors indicate higher NTD risk, while negative coefficients show lower NTD risk. The ordinary least squares (OLS) regression models in this study are built under the assumption of independence of subjects, but the cross-sectional survey has a hierarchical structure with respondents nested within survey clusters, which could potentially bias the standard errors. STATA 13 (StataCorp, College Station, TX, USA) SE, which has the capacity to address this problem, is used by imposing on our models a ‘cluster’ variable, that is, the identification numbers of respondents at the cluster level. This in turn adjusts the standard errors (SE) producing statistically robust parameter estimates.

## Results

### Historical Overview on the Distribution and Epidemiology of Neglected Tropical Diseases (NTDs) in Tanzania

Table 1 shows the various NTDs reported in Tanzania since the 19^th^ Century. So far, buruli ulcer, chagas disease and dracuncunliasis have never been reported in Tanzania. However, cholera, schistosomiasis (bilharzia), onchocerciasis (river blindness), trachoma (granular conjunctivitis), hookworm, whipworm, dengue fever, human African trypanosomiasis, leischmaniasis, leprosy and roundworm have been reported at least once during the last 200 years in Tanzania. Cholera outbreaks and cases surpass all cases and outbreaks of other NTDs in Tanzania. In general, prevalence and incidence of all NTDs have declined in the past 5 years in Tanzania. However, there are regional variations in the NTD type, number of cases and frequency of occurrence of the various NTDs.

**Table 1:** The distribution and epidemiology of Neglected Tropical Diseases (NTDs) in Tanzania.

S/N	Name of NTD	Reported in Tanzania						Prevalence since 1^st^ reported								Current prevalence Status
		Yes	No	Year (ref.)	Region	District	No. of cases in 1^st^ year (ref.)	Year	Cases (ref.)*	Region	District	Place of residence		Sex		
												Rural	Urban	M	F	
1	Buruli ulcer		√													
2	Chagas diseases		√													
3	Cholera	√		1821 (1)	1^st^ records	-	-	1977	1671 (4)							
								1977, 1978, 1981, 1983	(5, 6, 7)	TZ Mainland, Dar es Salaam						
								1986	(8)	Mara						
				1974 (2)	TZ Mainland	-	10 (2)	1992	18526 (2)							
								1996	1,100 (9)	Kigoma						
				1978 (3)	TZ Zanzibar	Tumbatu—Unguja	411 (3)	1997	40'249 (2, 10)	DSM	Illala, Kinondoni					
								1998		Mwanza, Tukwa						
								1999	11,855(11)							
								2002–2006	~2000 (2)	DSM, Dodoma, Kigoma, Lindi, Mbeya, Morogoro, Mtwara, Pwani, Tanga, Rukwa, Mwanza, Mara, Shinyanga, s						
								2006	8965 (2)	DSM						
									1507 (2)	Ruvuma						
									1030 (2)	Kigoma						
									315 (3)	Pemba, Unguja						
								2007	1092 (7)	Zanzibar						
								2008	500 (12, 13, 14, 15, 16, 17)	Arusha, Dar es Salaam, Kilimanajaro, Mara, Rukwa						
								2009–2010	211 (18)	Dar es Salaam						
									3454(19, 20)	Tanga						
									60 (21)	Mwanza						
									3 (22, 23, 24)	Mara						
								2013	300 (25)	Rukwa						
4	Dengue fever	√		1823 (26)	Zanzibar				7.7% (27)	Pemba						
				1870 (26)	Zanzibar				1.8% (27)	Iringa						
				2008 (5)	Kilimanjaro	Moshi	71 (5)									
5	Human African Trypanosomiasis			1902 (29) Gambiense												
		√		1910 (6) Rhodiense	Western, Northern and Southern Highlands			1919–1921(7,9)		Shinyanga	Maswa					
										Lindi	Kilwa	Luangwa				
								1925 (9), 1930, 1957, 1960 (9)		Lindi	Kilwa	Matandu				
										Kigoma, Tabora	Kasulu					
								1922 (7)			Ikoma					
								1925–1946 (8)	2119		Ikoma					
								1979–1992	6000 (29)	Tabora, Arusha, Kigoma, Rukwa	Kibondo, Kasulu, Sikonge, Mponda, Urambo					
6	Leischmaniasis	√		1964 (34)	Kilimanjaro		1									
7	Leprosy											√		55%	45%	0.85/10000 (35)
8	Dracuncunliasis		√													
9	Lymphatic filariasis (elephantiasis)	√		1911 (36)	Lindi		-	2008	3.3%							33/1000
					Mtwara		-	2008	3.3%							
					DSM		-	2008	3.3%							
					Tanga		-	2008	3.3%							
					Pwani		-	2008	3.3%							
					Morogoro		-	2008	-							
								1990 (37)	1.1%	Pemba						
								2000 (38)	28.5% males	Hale						
								2001	7.2%		Kwahani		√			
								1957 (39)	70% adult males		Kilwa					
					Lake Victoria Zone		-	2008	-							
					Lake Nyasa zone	Kyela,	-	2008	4.5%	Mbeya	Kyela,	√				
						Rungwe		2008	4.5%		Rungwe	√				
								2011 (40)	62.9% school children	Morogoro	Mvomero					
10	Schistosomiasis haematobium & mansoni (snail fever)	√		1895 (41,42)	Lake Victoria Zone		50%	1903 (43)	-	Zanzibar						51.5% (48)
								1961 (44)	203 [64%]	Mwanza	Usagara					
								1966 (45)	975 [42%]	Tabora	Bukumbi			664	311	
								1967 (46)	391 [65.2%]	Mwanza	Ukerewe					
								1969 (46)	614 [61.2%]	Zanzibar	Unguja					
								1983 (46)	730 [21%]	Morogoro	Ifakara					
								1985 (46)	483 [19.3%]	DSM						
								1997 (46)	2415 [67%]	Zanzibar	Pemba					
								2005 (46)	393 [62%]	Kilimanjaro	Mwanga					
								2008 (46)	1129 [78%]	Mwanza	Ilemela, Ukerewe					
								2009 (46)	311 [27.2%]	Tanga	Lushoto	Umba				
								2012 (47)	43 [18.1%]	Pwani	Mafia					
11	Onchocerciasis (river blindness)	√		1958	Tanga	Usambara	-	1984 (52)	22.7%	Tanga	Usambara					
								1990 (51)	58.6%	Morogoro	Mahenge	Bwakira				
									31.9%	Ruvuma						
									22.4%	Tanga	Amani					
									22.8%	Mbeya	Tukuyu					
								1991 (53)	60%	Central Tanzania						
								2000 (50)	120	Dodoma	Kongwa			32	88	25.4% (49)
									143		Mpwapwa			19	124	
									50	Dodoma	Dodoma			12	38	
									215		Manyoni			50	165	
12	Trachoma (eye infection)	√						2004(56)	13.9%	Kilimanjaro	Rombo	Kahe				
								2006 (55)	44%	Dodoma, Singida, Arusha, Mwanza, Shinyanga, Mtwara, Lindi, Coast and Tanga						
								2006 (54)	119 [9%]	Dodoma	Kongwa	√				
								2013 (57)	20.4%							20.4%
13	Hookworm;	√		1982–2007 (68)	URT		20–50%	1997 (60)	95%	Pemba						
								2000 (61)	72.5% school children	Mafia Island						
								2002 (62)	73.8% male 77.1% female	Tanga						
								2004 (63)	7.7% children	Unguja Island						
								2005 (58)	3145 (40%)	Shinyanga, Mwanza, Tabora and Mara						
								2007 (64)	32.9% pregnant women	Pemba Island						
								2007 (65)	11.9% school children	Zanzibar						
								2007 (66)	21.6% school children	Zanzibar						
								2009	548 [16.2%]	Zanzibar						
								2010 (59)	152 [38%]	Mwanza	Sengerema	Nyamatongo				
								2011 (40)	24.7% school children	Morogoro	Mvomero					
14	Roundworm	√		1977 (69)	URT		40%									
				1982–2007 (68)	URT		20–50%	1997 (60)	72%	Pemba						
								2009	548 [8%]	Zanzibar						
15	Whipworm	√		1982–2007 (68)	URT		20–50%	1997 (60)	96%	Pemba						
								2009 (67)	548 [62.8%]	Zanzibar						

* Frequencies [], References are shown in parentheses in columns 5, 8 and 10. All references in Table 1 are presented in S1 Text.

### Sample Characteristics

Each individual in the survey sample was simultaneously exposed to at least two NTDs. No individual was simultaneously exposed to four or more NTDs as shown in Table 2.

**Table 2:** Distribution of predictors by exposure to multiple NTDs in coastal Tanzania.

Variables	At least 2 NTDs	More than 2 NTDs	Chi-square & Cramér's V
**Compositional Attributes**			
***Biosocial factors***	%	%	
*Age*			
18–35	23.0	77.0	chi2(3) = 1.6642 Pr = 0.645 Cramér's V = 0.0364
36–50	22.9	77.1	
51–65	25.7	74.3	
More than 65	27.7	72.3	
*Sex (Gender)*			
Male	22.9	77.1	chi2(1) = 0.6513 Pr = 0.420 Cramér's V = -0.0228
Female	24.9	75.1	
*Ethnicity*			
Zaramo	65.3	34.7	chi2(2) = 292.1794 Pr = 0.000 Cramér's V = 0.4829
Sambaa	5.3	94.7	
Others	15.2	84.8	
***Socio-cultural factors***			
*Poverty*			
Poor	19.9	80.1	chi2(1) = 33.5928 Pr = 0.000 Cramér's V = -0.1637
Non-poor	36.1	63.9	
*Educational Attainment*			
No education	38.3	61.7	chi2(3) = 100.1362 Pr = 0.000 Cramér's V = 0.2827
Primary	33.7	66.3	
Secondary	14.9	85.1	
Tertiary	5.4	94.6	
*Employment*			
Unemployed	28.3	71.7	chi2(1) = 1.0168 Pr = 0.313 Cramér's V = 0.0285
Employed	23.6	76.4	
*Marital Status*			
Single	19.5	80.6	chi2(1) = 7.4346 Pr = 0.006 Cramér's V = -0.0770
Married	26.4	73.7	
**Contextual Attributes**			
*Region of Residence*			
Dar-es-Salaam	0.0	100.0	chi2(2) = 1.2e+03 Pr = 0.000 Cramér's V = 0.9978
Pwani	99.7	0.3	
Tanga	0.0	100.0	
*Self-reported household quality of life relative to others*			
The Worst	37.8	62.2	chi2(4) = 13.2716 Pr = 0.010 Cramér's V = 0.1029
Among the Worst	30.0	70.1	
About the Same	23.7	76.3	
Better	17.3	82.7	
Best in the Community	19.4	80.6	
*Residential Locality*			
*Rural*	52.4	47.7	chi2(1) = 381.2196 Pr = 0.000 Cramér's V = -0.5516
*Urban*	4.4	95.6	
*Residential Time*			
*Up to 5 years*	13.7	86.3	chi2(3) = 76.4820 Pr = 0.000 Cramér's V = 0.2471
*Up to 10 years*	19.5	80.5	
*Up to 15 years*	17.6	82.4	
*20 or more years*	38.2	61.8	
*Access to Health Services*			
*No access*	16.5	83.5	chi2(1) = 24.1026 Pr = 0.000 Cramér's V = -0.1387
*Access*	28.7	71.4	
*NTD Comorbidities*			
*0*	31.6	68.4	chi2(5) = 19.7724 Pr = 0.001 Cramér's V = 0.1257
*1*	17.9	82.1	
*2*	27.2	72.8	
*3*	24.2	75.8	
*4*	25	75	
*5 or more*	13.3	86.7	

About 75% of 1253 respondents were simultaneously exposed to three NTDs and 25% were exposed to 2 NTDs. Males and females were evenly distributed in terms of simultaneous exposures to 3 NTDs although females (54%) were more exposed to 2 NTDs than their male counterparts. NTD comorbidities was associated with gender (*x* ^2^(8) = 19.9265, pr = 0.011)). Also, there were regional variations in NTD comorbidities (*x* ^2^(16) = 37.4640, pr = 0.002)) and exposures to multiple NTDs (*x* ^2^(2) = 1.2e+03, pr = 0.000)). Although there are no statistically significant differences in NTD comorbidities according to poverty status there are differences in exposures to multiple NTDs by poverty status (*x* ^2^(1) = 33.5928, pr = 0.000)). In this study, the reported comorbidities were malaria (76%), hypertension (25%), tuberculosis (25%), HIV (10%), skin diseases (9%), pneumonia (6%), heart disease (5%), cholera (4%) diabetes (3%), hepatitis (2%) and cancer (1%). Based on the sample, malaria was the most common comorbidity. Based on the responses in the survey sample, inferential statistics (chi-square) did not find any statistically significant relationships between region of residence and each of the following diseases: hepatitis, skin diseases, pneumonia, HIV status, hypertension, cancer and heart disease. This means that these diseases were independent of region of residence. However, region was not independent of malaria (*x* ^2^(2) = 1.3e+03, Pr = 0.000), tuberculosis (*x* ^2^(2) = 5.9731, Pr = 0.045), cholera (*x* ^2^(2) = 19.1780, Pr = 0.000) and diabetes (*x* ^2^(2) = 11.7872, Pr = 0.003).

Exposures to multiple NTDs do not vary according to age even though NTD comorbidities vary by age of respondents. Both NTD comorbidities and exposures to multiple NTDs vary by educational attainment of respondents. In coastal areas of Tanzania, risks of cholera, hookworm and whipworm are higher in Dar es Salaam region than in Tanga and Pwani regions. Risks of schistosomiasis and trachoma are higher in Pwani and Tanga regions than in Dar es Salaam region. Risks of onchocerciasis are higher in Tanga region than in Pwani and Dar es Salaam regions. Based on the magnitude of Cramér's V, the strength of association of theoretically-relevant covariates and exposure to multiple NTDs in decreasing order of magnitude is as follows: region of residence > residential locality > ethnicity > educational attainment > residential time > poverty access to health services > NTD comorbidities > self-rated housing quality.

### Bivariate Analysis

Table 3 shows zero-order relationships between NTD risks and theoretically relevant covariates. All biosocial factors were significant predictors of NTD risk. Older individuals and females were associated with higher NTD risk scores. Age squared was statistically significant indicating possibly that the relationship between age and NTD risks was not linear. Each of the sociocultural factors was a significant predictor of NTD risks except employment (Table 3). Higher NTD comorbidities, higher exposures to multiple NTDs and higher poverty levels were each associated with higher NTD risks. Except residential time and self-reported household quality of life, all contextual factors including sources of drinking water in the wet and dry seasons, rural/urban status and region of residence were significant predictors of NTD risks.

**Table 3:** Bivariate relationships of risk of multiple exposures to neglected tropical diseases and explanatory variables (n = 1253) in coastal Tanzania.

Variables	Coefficient	Robust Std. Error	95% Confidence Interval
**Compositional Attributes**			
***Biosocial factors***			
Age	0.458***	0.090	-0.212–0.094
Sex (Gender)	0.588**	0.056	-0.202 0.019
Ethnicity	0.320**	0.106	0.111 0.529
Age Squared	0.102***	0.020	0.062 0.143
***Socio-cultural factors***			
Poverty	0.715***	0.056	-0.826–0.603
Educational Attainment	0.489***	0.027	0.436 0.542
Log income	0.369***	0.023	0.323 0.414
Employment	-0.109	0.106	-0.318 0.099
Type of current dwelling	-0.199***	0.017	-0.234–0.164
Marital Status	-0.237***	0.058	-0.352–0.121
**Contextual factors**			
Source of Drinking Water in Dry Season	0.249*	0.124	0.005 0.492
Source of Drinking Water in Wet Season	-0.345***	0.055	-0.453–0.236
Rural/Urban Status	-1.042***	0.168	-1.372–0.713
Residential Time	0.031	0.021	-0.271–0.187
Region of Residence	-1.111***	0.070	-1.109 0.170
Self-reported household quality of life relative to others	-0.096	0.128	-0.346 0.157
Access to Health Services	-0.447*	0.178	0.145 0.373
NTD Multiple Exposures	1.586***	0.166	1.260 1.911
NTD Comorbidities	2.765***	0.032	2.702 2.827

### Multivariate Analysis

Table 4 is a nested model that shows the multivariate relationship between NTD risks on the one hand and compositional and contextual factors, on the other hand. The statistically significant zero-order relationship between NTD risks, age, sex and ethnicity remains in the biosocial model in the multivariate analysis. The biosocial model explains only 4% of the variance in the relationship between NTD risks and theoretically relevant covariates. Females and older respondents were associated with higher NTD risks. When the model is adjusted for sociocultural factors, the original zero-order relationship between poverty and NTD risks unexpectedly disappears. Self-reported household quality of life relative to others was a significant predictor of NTD risks in the sociocultural model unlike educational attainment and marital status. The sociocultural model explains about 19% of the variance in the relationship between NTD risks and theoretically relevant covariates.

**Table 4:** Compositional and contextual determinants of risk of neglected tropical diseases (n = 1253) in coastal Tanzania.

	Compositional Factors					Contextual factors			
	Model 1: Biosocial factors			Model 2: Socio-cultural factors		Model 3: Place & Neighbourhood factors			
	Coef.	Beta Coef.	Std. Err.	Coef.	Beta Coef.	Std. Err.	Coef.	Beta Coef.	Std. Err.
***Intercept***	1.13**	-	0.43	1.46	-	0.69	0.19	-	0.19
Gender	0.68***	0.11	0.17	0.72***	0.12	0.18	0.03	0.005	0.04
Age	0.51***	0.15	0.09	0.56***	0.17	0.09	0.04Ψ	0.013	0.03
Ethnicity	0.35**	0.13	0.10	0.29*	0.07	0.10	0.21***	0.055	0.02
Poverty				0.35	0.05	0.21	0.11*	0.015	0.05
Educational Attainment				0.18	0.05	0.12	0.05***	0.14	0.03
Marital Status				-0.23	-0.12	0.18	0.03	0.004	0.04
Employment Status				-0.75*	-0.06	0.35	0.11	0.096	0.08
Self-reported household quality of life relative to others							0.03	0.066	0.03
Access to Health Services							-0.29***	-0.046	0.04
Residential Locality							-0.69***	-0.109	0.05
Source of Drinking Water in Wet Season							-0.02	-0.005	0.02
Source of Drinking Water in Dry Season							0.01	0.003	0.01
NTD Comorbidities							2.74**	0.960	0.03
**R** ^**2**^	**0.04**			**0.23**			**0.95**		

In the place and neighbourhood model when both compositional and contextual factors (collective effect) are taken into account some interesting results emerge. For instance, the relationship between poverty and NTD risks which disappeared in the sociocultural model re-appears. The statistical significance of the relationship between ethnicity and NTD risks, which reduced in the sociocultural model, also becomes stronger in the neighbourhood model. In the sociocultural model, educational attainment was not a significant predictor of NTD risks unlike in the neighbourhood model. Employment status became statistically insignificant when collective effect is controlled. Access to health services, residential locality and NTD comorbidities were significant predictors of NTD risks when collective effect was accounted for in Table 3 unlike self-reported household quality of life and sources of drinking water in wet and dry seasons. Based on the standardised regression coefficients of non-binary predictors in the place and neighbourhood model, the effect size in decreasing order of magnitude is as follows: NTD morbidities > educational attainment > self-reported household quality of life > ethnicity. The place and neighbourhood model in the multivariate analysis explains 72% of the variance in the relationship between NTD risks and theoretically relevant covariates.

Poorer, uneducated, unemployed individuals had higher NTD risk scores compared to their relatively affluent, educated and employed counterparts. This implies hypothesis 1, which suggests that poorer individuals will be associated with higher exposures to multiple NTDs and by extension experience higher NTD risks than their less poor counterparts cannot be rejected. Individuals living in poorer neighbourhoods with limited access to health, social, water and sanitation services had higher NTD risk scores than their counterparts living in less-deprived neighbourhoods implying that hypothesis 2 cannot also be rejected. However, on disaggregating the results by gender, we did not find any evidence to support the third hypothesis which posits that women, by dint of social disadvantage, are more exposed to multiple NTDs and higher NTD comorbidities and so experience higher NTD risks than their male counterparts. In the females’ model, poverty was not even a significant predictor of NTD risks. Only, ethnicity, access to health services, rural/urban status and NTD comorbidities were significant predictors of NTD risks for women. In the males model however, poverty, age, ethnicity, educational attainment, employment status, access to health services and NTD comorbidities were significant predictors of NTD risks. Poorer older males without access to health services in rural areas had higher NTD risks compared to less poor younger males with access to health services living in urban areas.

## Discussion

In this paper, we present a historical overview on the distribution of NTDs in Tanzania during the last two centuries. We also examined the effect sizes of the compositional and contextual determinants of NTD risks with emphasis on coastal Tanzania.

We found regional variations in the type, number of cases and frequency of occurrence of the various NTDs. This is expected as a defining characteristic of most NTDs is their locality [5, 13]. This implies that NTD-induced morbidity and mortality may vary considerably geographically due to different local factors [5]. This result is not entirely surprising given regional and national policy initiatives and clinical interventions on NTDs in Tanzania. To date, Tanzania’s NTD program has distributed over 81 million NTD treatments to approximately 34 million people with USAID support [25]. Tanzania has also established an extensive monitoring and evaluation system, including a national NTD database, to gauge the national program’s progress towards NTD control and elimination. With support from USAID, tablet computers are being used to document the burden of trachoma. Also, real-time results of the data collected each day are instantly available online for quick analysis and decision-making. Furthermore, Tanzania has integrated data collection for lymphatic filariasis [26, 27, 28], soil-transmitted helminthes, and schistosomiasis as is a cost effective mechanism to monitor the impact of NTD control activities.

The finding that NTD risks are unequally distributed over geographic space has several important implications. First, it suggests that localities of high burden of NTDs are likely to diminish within statistical averages at higher (regional or national) levels. Second, it presupposes that curative or preventive interventions will become more efficient provided they can be focused on the localities, particularly as populations at these locations are likely to be burdened by several NTDs at the same time. In this sense, exposure and comorbidities offer valuable opportunities for integrated control of the various NTDs in Tanzania. Also, from a sustainability perspective it is imperative to continue targeting the most affected populations in order to reduce the underlying health inequalities that may be driving NTD prevalence among disadvantaged groups [29]. In fact, the mass drug administration approach in Tanzania appears to be doing this. For some, the treatments go to a more focal level depending on disease prevalence (e.g., STH, schistosomiasis, onchocerciasis), while for others (trachoma, lymphatic filariasis) the health district is the implementation unit.

This view is reinforced by the positive relationship (direct) between poverty and NTD risks. Furthermore, poor neighbourhoods, which are characterised by the lack of or limited access to health, social, water and sanitation services, were associated with higher NTD risks. These results are consistent with previous scholarly work on NTDs. For instance, Aagaard-Hansen and Chaignat [5] investigated inequities and social determinants of NTDs and found that of all the predictors in their study, poverty (lack of purchasing power) is the only factor having recognized relationship to all 13 NTDs. They proposed two main mechanisms by which poverty mutually reinforces NTDs. They explained that poverty is a structural social determinant and is intrinsically connected to the intermediate determinants of water, sanitation, housing and clustering. Furthermore, poverty may actually be a consequence of some of the NTDs (for example buruli ulcer, dengue fever, human African trypanosomiasis, leishmaniasis and lymphatic filariasis)–either as a result of very expensive treatment [30], or indirectly through loss of labour capability [5]. In terms of housing, there are clear relationships between housing quality, access to water and sanitation services, clustering and neighbourhood quality of life all of which contribute to differential exposure to NTDs. For instance, the defining characteristics of poor neighbourhoods include low quality housing, clustering and limited access to water, and sanitation and health services [30–33].

All respondents were simultaneously exposed to at least 2 NTDs and there were regional and poverty-related differentials in exposure to NTDs. This finding is consistent with previous studies on NTDs. According to Blas and Kurup [34], the same level of exposure may have different effects on different socioeconomic groups, depending on their social, cultural and economic environments and cumulative life course factors. In this situation, clustering of risk factors in some population groups, such as social exclusion, low income, overcrowded housing and poor access to health services, may be as important as the individual exposure itself [34].

Source of drinking water in the wet season, access to health services and good housing quality were each negatively (inversely) associated with NTD risks. The fact that poorer, uneducated, unemployed individuals had higher NTD risk scores compared to their relatively affluent, educated and employed counterparts likely suggests a social gradient in NTD risks in coastal Tanzania. The social gradient of NTD risks could possibly emanate from multiple interacting factors, including socio-environmental risk factors, social determinants, comorbid conditions, general health status, health-seeking behaviours and access to NTD health care services.

Exposures to multiple NTDs and NTD comorbidities (co-occurrence of one or more diseases or disorders in an individual) were both positively associated with NTD risks. Similarly, age was robustly associated with higher risks of NTDs. It is argued that comorbidity is related to poorer health outcomes, more complex disease management, and increased health care costs [35]. According to Degenhardt et al. [36] several hypotheses exist concerning the reasons why comorbidity (e.g., in the context of NTDs) might occur, including that: (a) there is a causal relationship between the comorbidity and NTD type; (b) that common factors increase the likelihood of both NTDs and comorbidities; and (c) that the relationship is spurious (artefactual), resulting from factors such as the methods with which the sample was selected. Comorbidity potentially has implications for theories of aetiology and distribution of neglected tropical diseases. For instance, if comorbidity arises because different health problems (e.g. malaria, pneumonia, etc.) share the same risk factors, then interventions addressing these risk factors should reduce the prevalence of these multiple problems [36].

We did not find evidence that women are more exposed to multiple NTDs and have higher NTD comorbidities and so experience higher NTD risks than their male counterparts. However, several studies highlight gender differentials in exposure to NTDs and risks of NTDs. For instance, Aagaard-Hansen and Chaignat [5] suggest there is substantial difference in morbidity and mortality rates for males and females by neglected tropical disease. Cattand et al. [37] also observed that, for human African trypanosomiasis, men are affected at nearly twice the rate of women. Thus, males are disproportionately affected by human African trypanosomiasis and schistosomiasis due to exposure, whereas women suffer more from leprosy (stigma) and trachoma (blindness). The relationship between ethnicity and NTD risks was robust and remained significant even when compositional and contextual factors were accounted for in the multivariate model. This result supports studies which show association between prevalence of dracunculiasis and particular ethnic groups [38]. Ethnic differences may be attributable to different sociocultural and behavioural practices. For instance, people of diverse cultural backgrounds often make different attributions of illness, health, disease, symptoms and treatment [39]. Afrocentric societies usually make spiritual attributions to ill-health compared to Eurocentric societies [37]. In this sense, indigenous beliefs and practices, which are rooted in specific cultures, might affect health outcomes (NTD risks) and interactions with the NTD prevention and treatment services provided in the health care setting.

This study is not without limitations. First, it focused exclusively on coastal Tanzania although NTDs transcend this geographic region. Perhaps, future research could elucidate NTD risks in both coastal and non-coastal regions in order to obtain a much more comprehensive representation of NTD risks across Tanzania. This study is partly based on a cross-sectional survey, which misses the temporal dimension of NTD risks. Given that NTD risks may vary systematically with time it is imperative to design future studies that take into account both the spatial and temporal aspects of NTD risks.

### Conclusion

The study also presented a brief historical perspective on the distribution of NTDs in Tanzania during the last two centuries. This study also assessed exposure to multiple NTD risks using a cross sectional of 1253 individuals in coastal Tanzania. Both compositional and contextual factors act in complex ways to give rise to NTD risks. In particular, low socioeconomic status, poor neighbourhoods, age, NTD comorbidities and exposures to multiple NTDs are robustly associated with an increased risk of NTDs in coastal Tanzania. The link between poverty and NTD risks is persistent and remains even when compositional and contextual factors are adjusted. Based on the standardised regression coefficients of non-binary predictors in the place and neighbourhood model, the effect size in decreasing order of magnitude (importance) is as follows: NTD morbidities > educational attainment > self-reported household quality of life > ethnicity. On the whole, based on the findings, contextual factors are more important practically than compositional factors in terms of relative contribution to NTDs risks. Contextual factors cumulatively explained 72% of the variance in NTD risks whereas compositional (biosocial and sociocultural) factors jointly explained only 23% of the variance. Given the plethora of sociopolitical, economic and cultural factors that culminate in inequities in NTD exposures, comorbidities and risks between poor and less deprived individuals and groups an integrated approach to addressing NTD risks is warranted.

## Supporting Information

S1 Text List of references in Table 1. (DOCX)

S1 Checklist STROBE statement-checklist of items included in our study. (DOC)
