# Correction: Historical Perspective and Risk of Multiple Neglected Tropical Diseases in Coastal Tanzania: Compositional and Contextual Determinants of Disease Risk

**DOI:** 10.1371/journal.pntd.0004048

**Published:** 2015-09-17

**Authors:** The PLOS Neglected Tropical Diseases Staff

The date this article was received is listed incorrectly. The correct date received is the following: October 26, 2014.
