# Supplementary material for: Historical Perspective and Risk of Multiple Neglected Tropical Diseases in Coastal Tanzania: Compositional and Contextual Determinants of Disease Risk
Source: PLoS Negl Trop Dis. 2015 Aug 4;9(8):e0003939. doi: 10.1371/journal.pntd.0003939 (PMC4524715; doi:10.1371/journal.pntd.0003939)
Supplement: S1 Text — (DOCX) [file pntd.0003939.s001.docx]

**SI Text: List of references in Table 1**

1. Pollitzer, R. (Ed.) (1959). Cholera, WHO Monograph Series Number 43. World Health Organization, Geneva; Switzerland

2. WHO (2008). Global Task Force on Cholera Control. Cholera Country Profile: United Republic of Tanzania. 7 April, 2008 3.WHO, 2006 Global Task Force on Cholera Control. Cholera Country Profile: Zanzibar, Tanzania, 2006

4. Mhalu FS, Mwaluko GMP, Kilama WL, Mandara MP, Muru M, Marcpherson CNL (1991). Health and Disease in Tanzania. London: Harper Collins Academic. p. 46-55

5. Mhalu, F. S., Mmari, P. W., & Ijumba, J. (1979). Rapid emergence of El Tor vibrio cholera resistant to antimicrobial agents during first six months of fourth cholera epidemic in Tanzania. *The Lancet*, *313*(8112), 345-347

6. Mandara, M. P., & Mhalu, F. S. (1980). Cholera control in an inaccessible district in Tanzania: importance of temporary rural centres. *Medical journal of Zambia*, *15*(1), 10-13

7. Mhalu, F. S., Mtango, F. D. E., & Msengi, A. E. (1984). Hospital outbreaks of cholera transmitted through close person-to-person contact. *The Lancet*, *324*(8394), 82-84

8. Killewo, J. Z., Amsi, D. M. D., & MHALU, F. S. (1989). An investigation of a cholera epidemic in Butiama village of the Mara region, Tanzania. *Journal of diarrhoeal diseases research*, 13-17

9. ProMED-mail. Cholera, Refugees - Tanzania from Zaire. ProMED-mail 1996; 09 Dec: 19961209.2034. International Society for Infectious Diseases. Assessed on 18^th^ June 2014 from <http://www.promedmail.org/direct.php?id=19961209.2034>

10. Acosta, C. J., Galindo, C. M., Kimario, J., Senkoro, K., Urassa, H., Casals, C., Corachán, M.,... & Alonso, P. L. (2001). Cholera outbreak in southern Tanzania: risk factors and patterns of transmission. *Emerging infectious diseases*, *7*(3 Suppl), 583

11. Urassa, W. K., Mhando, Y. B., Mhalu, F. A., & Mgonja, S. J. (2000). Antimicrobial susceptibility pattern of Vibrio cholerae 01 strains during two cholera outbreaks in Dar Es Salaam, Tanzania. *East African medical journal*, *77*(7)

12. ProMED-mail. Cholera, Diarrheoa & Dysentery update 2008 (09). ProMED-mail 2008; 06 Feb: 20080206.0486. International Society for Infectious Diseases. Accessed on 18^th^ June 2014 from <http://www.promedmail.org/direct.php?id=20080206.0486>

13. ProMED-mail. Cholera, Diarrheoa & Dysentery update 2008 (25). ProMED-mail 2008; 08 May: 20080508.1572. International Society for Infectious Diseases. Accessed on 18^th^ June 2014 from <http://www.promedmail.org/direct.php?id=20080508.1572>

14. ProMED-mail. Cholera, Diarrheoa & Dysentery update 2008 (23). ProMED-mail 2008; 18 Apr: 20080418.1394. International Society for Infectious Diseases. Accessed on 18^th^ June 2014 from <http://www.promedmail.org/direct.php?id=20080418.1394>

15. ProMED-mail. Cholera, Diarrheoa & Dysentery update 2008 (36). ProMED-mail 2008; 15 Sep: 20080915.2882. International Society for Infectious Diseases. Accessed on 18^th^ June 2014 from <http://www.promedmail.org/direct.php?id=20080915.2882>

16. ProMED-mail. Cholera, Diarrheoa & Dysentery update 2008 (24).ProMED-mail 2008; 25 Apr: 20080425.1446. International Society for Infectious Diseases. Accessed on 18^th^ June 2014 from <http://www.promedmail.org/direct.php?id=20080425.1446>

17. ProMED-mail. Cholera, Diarrheoa & Dysentery update 2008 (40). ProMED-mail 2008; 14 Oct: 20081014.3253. International Society for Infectious Diseases. Accessed on 18^th^ June 2014 from <http://www.promedmail.org/direct.php?id=20081014.3253>

18. ProMED-mail. Cholera, Diarrheoa & Dysentery update 2009 (30). ProMED-mail 2009; 25 Nov: 20091125.4044. International Society for Infectious Diseases. Accessed on 18^th^ June 2014 from <http://www.promedmail.org/direct.php?id=20091125.4044>

19. ProMED-mail. Cholera, Diarrheoa & Dysentery update 2009 (25). ProMED-mail 2009; 09 Oct: 20091009.3500. International Society for Infectious Diseases. Accessed on 18^th^ June 2014 from <http://www.promedmail.org/direct.php?id=20091009.3500>

20. ProMED-mail. Cholera, Diarrheoa & Dysentery update 2009 (27). ProMED-mail 2009; 03 Nov: 20091103.3795. International Society for Infectious Diseases. Accessed on 18^th^ June 2014 from <http://www.promedmail.org/direct.php?id=20091103.3795>

21. ProMED-mail. Cholera, Diarrheoa & Dysentery update 2010 (02): Africa. ProMED-mail 2010; 08 Feb: 20100208.0428. International Society for Infectious Diseases. Accessed on 18^th^ June 2014 from <http://www.promedmail.org/direct.php?id=20100208.0428>

22. Khatib, A. M., Ali, M., von Seidlein, L., Kim, D. R., Hashim, R., Reyburn, R., Ley, B., ... & Deen, J. (2012). Effectiveness of an oral cholera vaccine in Zanzibar: findings from a mass vaccination campaign and observational cohort study. *The Lancet infectious diseases*, *12*(11), 837-844

23. ProMED-mail. Cholera, Diarrheoa & Dysentery update 2009 (05). ProMED-mail 2009; 12 Jan: 20090112.0124. International Society for Infectious Diseases. Accessed on 18^th^ June 2014 from <http://www.promedmail.org/direct.php?id=20090112.0124> 24. ProMED-mail. Cholera, Diarrheoa & Dysentery update 2010 (30). ProMED-mail 2010; 31 Dec: 20101231.4608. International Society for Infectious Diseases. Accessed on 18^th^ June 2014 from <http://www.promedmail.org/direct.php?id=20101231.4608> 25. ProMED-mail. Cholera, Diarrheoa & Dysentery update 2013 (08): Africa and Asia. ProMED-mail 2013; 31 Jan: 20130131.1522070. International Society for Infectious Diseases. Accessed on 18^th^ June 2014 from <http://www.promedmail.org/direct.php?id=20130131.1522070>

26. Carey, D. E. (1971). Chikungunya and dengue: a case of mistaken identity? *Journal of the History of Medicine and Allied Sciences*, *26*(3), 243-262

27. Berger, S. (2013). Infectious Diseases of Tanzania. GIDEON Informatics Inc. Retrieved on 30^th^ December 2013 from <http://web.gideononline.com/web/epidemiology/index.php?disease=10590&country=G278&view=Distribution>

28. Hertz, J. T., Munishi, O. M., Ooi, E. E., Howe, S., Lim, W. Y., Chow, A., ... & Crump, J. A. (2012). Chikungunya and dengue fever among hospitalized febrile patients in northern Tanzania. *The American journal of tropical medicine and hygiene*, *86*(1), 171-177.

29. Migchelsen, S. J., Büscher, P., Hoepelman, A. I., Schallig, H. D., & Adams, E. R. (2011). Human African trypanosomiasis: a review of non-endemic cases in the past 20 years. *International Journal of Infectious Diseases*, *15*(8), e517-e524

30. Kilonzo, B. S., & Komba, E. K. (1993). The current epidemiology and control of trypanosomiasis and other zoonoses in Tanzania. *The Central African journal of medicine*, *39*(1), 10-20.

31. Davey, JB (1924) The outbreak of human trypanosomosis (Trypanosoma rhodesiense infection) in Mwanza district, Tanganyika Territory. Transactions of the Royal Society of Tropical. Medicine and Hygiene 17, 474-482.

32. Fairbairn, H. (1948). Sleeping sickness in Tanganyika territory, 1922–1946. *Tropical Diseases Bulletin*, *45*(1), 1-17.

33. Hide, G. (1999). History of sleeping sickness in East Africa. *Clinical microbiology reviews*, *12*(1), 112-125.

34. Andersen, O. S. (1964). Dermal leishmaniasis in a patient with leprosy in western Tanganyika. *East African medical journal*, *41*, 471.

35. National TB & Leprosy Program (NTLP) Strategic Plan 2009/2010 ‐ 2015/2016. Retrieved on 1^st^ April 2014 from <http://ntlp.go.tz/index.php?option=com_phocadownload&view=category&download=2:strategic-plan&id=3:plans&Itemid=139>

36. Malecela, M. N., Kilima, P., & Mackenzie, C. D. (2008). Implementation and management of lymphatic filariasis control and elimination programmes: the Tanzanian experience. *Lymphatic filariasis research and control in Eastern and Southern Africa*. p.112-123

37. Pedersen, E. M., Kilama, W. L., Swai, A. B. M., Kihamia, C. M., Rwiza, H., & Kisumku, U. M. (1999). Bancroftian filariasis on Pemba Island, Zanzibar, Tanzania: An update on the status in urban and semi‐urban communities. *Tropical Medicine & International Health*, *4*(4), 295-301

38. Massaga, J. J., Salum, F. M., & Savael, Z. X. (2000). Clinical and parasitological aspects of Bancroftian filariasis in Hale, northeast Tanzania. *The Central African journal of medicine*, *46*(9), 237-241

39. Nielsen, N. O., Simonsen, P. E., Magnussen, P., Magesa, S., & Friis, H. (2006). Cross-sectional relationship between HIV, lymphatic filariasis and other parasitic infections in adults in coastal northeastern Tanzania. *Transactions of the Royal Society of Tropical Medicine and Hygiene*, *100*(6), 543-550

40. Mboera, L. E., Senkoro, K. P., Rumisha, S. F., Mayala, B. K., Shayo, E. H., & Mlozi, M. R. (2011). < i> Plasmodium falciparum</i> and helminth coinfections among schoolchildren in relation to agro-ecosystems in Mvomero District, Tanzania. *Acta tropica*, *120*(1), 95-102

41. Doumenge, J.P., Mott, K.E., Cheung, C., Villenave, D., Chapuis, O., Perrin, M.F., Reaud-Thomas, G., 1987. Atlas of the global distribution of schistosomiasis. Talence, CEGET-CNRS, Geneva, WHO

42. Cook, J.H. (1909). Distribution of Bilhaziasis on the Victoria Nyanza. British Medical Journal 1, 1356

43. Aders, W.M. (1928). Schistosomiasis prevalence and malacological survey in Zanzibar and Pemba Islands. In Annual Report on the medical, sanitary and Biological Division for Zanzibar Protectorate.: British Empire; 1928

44. Jordan, P. (1961). Schistosoma haematobium infection in a Sukuma Village, Tanganyika. *Bulletin of the World Health Organization*, *25*(4-5), 695

45. Forsyth, D. M., & Bradley, D. J. (1966). The consequences of bilharziasis: Medical and public health importance in north-west Tanzania. *Bulletin of the World Health Organization*, *34*(5), 715.

46. Mazigo, H. D., Nuwaha, F., Kinung’hi, S. M., Morona, D., de Moira, A. P., Wilson, S., ... & Dunne, D. W. (2012). Epidemiology and control of human schistosomiasis in Tanzania. *Parasit Vectors*, *5*, 274.

47. Stothard, J. R., Ameri, H., Khamis, I., Blair, L., Nyandindi, U. S., Kane, R. A., ... & Rollinson, D. (2013). Parasitological and malacological surveys reveal urogenital schistosomiasis on Mafia Island, Tanzania to be an imported infection. *Acta tropica*, *128*(2), 326-333.

48. Rollinson, D., Knopp, S., Levitz, S., Stothard, J. R., Tchuem Tchuenté, L. A., Garba, A., ... & Utzinger, J. (2013). Time to set the agenda for schistosomiasis elimination. *Acta tropica*, *128*(2), 423-440.

49. Masesa, D. E., Moshiro, C., Masanja, H., Mkocha, H., Ngirwamungu, E., Kilima, P., ... & Saguti, G. (2013). Prevalence of active trachoma in Tanzania. *JOECSA*, *13*(3).

50. West, S., Nguyen, M. P., Mkocha, H., Holdsworth, G., Ngirwamungu, E., Kilima, P., & Munoz, B. (2004). Gender equity and trichiasis surgery in the Vietnam and Tanzania national trachoma control programmes. *British journal of ophthalmology*, *88*(11), 1368-1371.

51. Mweya, C. N., Kalinga, A. K., Kabula, B., Malley, K. D., Ruhiso, M., & Maegga, B. T. (2008). Onchocerciasis situation in the Tukuyu focus of southwest Tanzania after ten years of ivermectin mass treatment. *Tanzania Journal of Health Research*, *9*(3), 174-179.

52. Muro, A. I., & Mziray, N. R. (1989). Decline in onchocerciasis in the eastern Usambara mountains, north eastern Tanzania, and its possible relationship to deforestation. *Acta Leidensia*, *59*(1-2), 141-150.

53. West, S. K., Munoz, B., Turner, V. M., Mmbaga, B. B. O., & Taylor, H. R. (1991). The epidemiology of trachoma in central Tanzania. *International Journal of Epidemiology*, *20*(4), 1088-1092.

54. West, S. K., Emerson, P. M., Mkocha, H., Mchiwa, W., Munoz, B., Bailey, R., & Mabey, D. (2006). Intensive insecticide spraying for fly control after mass antibiotic treatment for trachoma in a hyperendemic setting: a randomised trial. *The Lancet*, *368*(9535), 596-600.

55. Tanzania Society for the Blind (2013). Trachoma escalates despite sustained remedial crusade. Retrieved on 31^st^ December 2013 from <http://www.tsb.or.tz/index.php/highlights/more/trachoma_escalates_despite_sustained_remedial_crusade/>

56. Solomon, A. W., Holland, M. J., Alexander, N. D., Massae, P. A., Aguirre, A., Natividad-Sancho, A., ... & Mabey, D. C. (2004). Mass treatment with single-dose azithromycin for trachoma. *New England Journal of Medicine*, *351*(19), 1962-1971.

57. Hu, V. H., Holland, M. J., & Burton, M. J. (2013). Trachoma: protective and pathogenic ocular immune responses to Chlamydia trachomatis. *PLoS neglected tropical diseases*, *7*(2), e2020.

58. London Imperial College (2014). Schistosoma Control Initiative. Retrieved on 1^st^ April 2014 from <http://www3.imperial.ac.uk/schisto/wherewework/tanzania/tanzaniaimpact>

59. Mazigo, H. D., Waihenya, R., Lwambo, N. J., Mnyone, L. L., Mahande, A. M., Seni, J., ... & Mkoji, G. M. (2010). Research Co-infections with Plasmodium falciparum, Schistosoma mansoni and intestinal helminths among schoolchildren in endemic areas of northwestern Tanzania. Parasites & Vectors 2010, 3:44.

60. Albonico, M., Chwaya, H. M., Montresor, A., Stolfzfus, R. J., Tielsch, J. M., Alawi, K. S., & Savioli, L. (1997). Parasitic infections in Pemba Island school children. *East African medical journal*, *74*(5), 294-298.

61. Albonico, M., Ramsan, M., Wright, V., Jape, K., Haji, H. J., Taylor, M., ... & Bickle, Q. (2002). Soil-transmitted nematode infections and mebendazole treatment in Mafia Island schoolchildren. *Annals of tropical medicine and parasitology*, *96*(7), 717-726.

62. Nielsen, N. O., Simonsen, P. E., Magnussen, P., Magesa, S., & Friis, H. (2006). Cross-sectional relationship between HIV, lymphatic filariasis and other parasitic infections in adults in coastal northeastern Tanzania. *Transactions of the Royal Society of Tropical Medicine and Hygiene*, *100*(6), 543-550.

63. Stothard, J. R., French, M. D., Khamis, I., Basáñez, M. G., & Rollinson, D. (2009). The epidemiology and control of urinary schistosomiasis and soil-transmitted helminthiasis in schoolchildren on Unguja Island, Zanzibar. *Transactions of the Royal Society of Tropical Medicine and Hygiene*, *103*(10), 1031-1044.

64. Young, S. L., Goodman, D., Farag, T. H., Ali, S. M., Khatib, M. R., Khalfan, S. S., ... & Stoltzfus, R. J. (2007). Geophagia is not associated with Trichuris or hookworm transmission in Zanzibar, Tanzania. *Transactions of the Royal Society of Tropical Medicine and Hygiene*, *101*(8), 766-772.

65. Knopp, S., Khalfan, A. M., Khamis, I., Mgeni, A. F., Stothard, J. R., Rollinson, D., ... & Utzinger, J. (2008). Spatial distribution of soil-transmitted helminths, including Strongyloides stercoralis, among children in Zanzibar. *Geospatial health*, *3*(1), 47-56.

66. Knopp, S., Mohammed, K. A., Rollinson, D., Stothard, J. R., Khamis, I. S., Utzinger, J., & Marti, H. (2009). Changing patterns of soil-transmitted helminthiases in Zanzibar in the context of national helminth control programs. *The American journal of tropical medicine and hygiene*, *81*(6), 1071-1078.

67. KPFE (Commission for Research Partnerships with Development Countries) (2009). Efficacy of albendazole and mebendazole alone or in combination with ivermectin against Trichuris trichiura and other soil-transmitted helminths in schoolchildren in Zanzibar, Tanzania. Retrieved on 1^st^ April 2013 from <http://www.kfpe.ch/projects/jeuneschercheurs/knopp.php>

68. Global Atlas of Helminthes Infections (GAHI) (2014). Retrieved on 1^st^ June 2014 from <http://www.thiswormyworld.org/>. 69. Carswell, F., Merrett, J., Merrett, T. G., Meakins, R. H., & Harland, P. S. E. G. (1977). IgE, parasites and asthma in Tanzanian children. *Clinical & Experimental Allergy*, *7*(5), 445-453.
